# Investigating potentially salvageable penumbra tissue in an in vivo model of transient ischemic stroke using sodium, diffusion, and perfusion magnetic resonance imaging

**DOI:** 10.1186/s12868-016-0316-1

**Published:** 2016-12-07

**Authors:** Friedrich Wetterling, Eva Chatzikonstantinou, Laurent Tritschler, Stephen Meairs, Marc Fatar, Lothar R. Schad, Saema Ansar

**Affiliations:** 1 Computer Assisted Clinical Medicine, Heidelberg University, Mannheim, Germany; 2 Trinity Institute of Neuroscience, University of Dublin, Dublin, Ireland; 3 Department of Neurology, Universitätsmedizin Mannheim, Heidelberg University, Mannheim, Germany; 4 CESP, INSERM UMRS1178, Faculté de Pharmacie, University Paris-Sud, Université Paris-Saclay, 92296 Chatenay-Malabry, France; 5 Division of Experimental Vascular Research, Department of Clinical Sciences, Lund University, Sölvegatan 17, BMC A13, 22184 Lund, Sweden

**Keywords:** Diffusion, Magnetic resonance imaging, Perfusion, Sodium, Stroke

## Abstract

**Background:**

Diffusion magnetic resonance imaging (MRI) is the current-state-of-the-art technique to clinically investigate acute (0–24 h) ischemic stroke tissue. However, reduced apparent diffusion coefficient (ADC)—considered a marker of tissue damage—was observed to reverse spontaneously during the subacute stroke phase (24–72 h) which means that low ADC cannot be used to reflect the damaged tissue after 24 h in experimental and clinical studies. One reason for the change in ADC is that ADC values drop with cytotoxic edema (acute phase) and rise when vasogenic edema begins (subacute phase). Recently, combined ^1^H- and ^23^Na-MRI was proposed as a more accurate approach to improve delineation between reversible (penumbra) and irreversible ischemic injury (core). The aim of this study was to investigate the effects of reperfusion on the ADC and the sodium MRI signal after experimental ischemic stroke in rats in well-defined areas of different viability levels of the cerebral lesion, i.e. core and penumbra as defined via perfusion and histology. Transient middle cerebral artery occlusion was induced in male rats by using the intraluminal filament technique. MRI sodium, perfusion and diffusion measurement was recorded before reperfusion, shortly after reperfusion and 24 h after reperfusion. The animals were reperfused after 90 min of ischemia.

**Results:**

Sodium signal in core did not change before reperfusion, increased after reperfusion while sodium signal in penumbra was significantly reduced before reperfusion, but showed no changes after reperfusion compared to control. The ADC was significantly decreased in core tissue at all three time points compared to contralateral side. This decrease recovered above commonly applied viability thresholds in the core after 24 h.

**Conclusions:**

Reduced sodium-MRI signal in conjunction with reduced ADC can serve as a viability marker for penumbra detection and complement hydrogen diffusion- and perfusion-MRI in order to facilitate time-independent assessment of tissue fate and cellular bioenergetics failure in stroke patients.

## Background

Stroke is the third leading cause of death worldwide. Overall, current stroke treatments, including use of endovascular therapy or embolectomy, have only a minor impact on this major public health problem [1]. Use of tissue plasminogen activators, the primary drug option, is limited by a narrow time window (0–4.5 h post stroke) and the need to first diagnose the stroke, as the drugs help in ischemic stroke but are detrimental in hemorrhagic stroke [2]. The number of stroke patients who actually receive these drugs is around 25% of stroke patients in tertiary stroke centers, mainly due to factors such as unknown stroke onset time, the narrow time window and the high number of exclusion criteria for currently approved treatments. Hence, it is of significant importance to enable selecting patients that can potentially benefit from thrombolytic treatment with unknown onset time.

Magnetic resonance imaging (MRI) is an important diagnostic tool for accurate diagnosis of stroke and evaluation of risks and benefits of thrombolysis. It is thought that determining hypoperfused, but potentially salvageable penumbral tissue can aid to find stroke patients who will respond well to thrombolysis. Furthermore, knowledge of the location and extent of the penumbra will help confirming the effect of potential new treatments in future clinical trials. Indeed, thrombolytic treatment outcomes are more successful when patients underwent an MRI diagnosis [3]. Perfusion diffusion mismatch is the MRI technique of choice currently available for assessing the amount of potentially salvageable tissue where the mismatch between the ischemically injured tissue is identified using diffusion MRI and the hypoperfused tissue volume is determined using standard ^1^H-MRI [4]. The overall aim of this approach is to detect the amount of still viable penumbra tissue, which may be at risk of infarction, if the tissue remains hypoperfused due to compromised supply of blood to this brain region. This is an indirect approach to identify penumbra leading to the assumption that low diffusion capability of hydrogen nuclei indicates permanently damaged tissue. Hence, this rather conservative approach leads to a lack of accuracy in the determining the volumetric ratio of still-viable to permanently damaged tissue. Several recent studies reported that the perfusion–diffusion mismatch does not accurately outline the ischemic penumbra. In fact, the penumbra appears to be underestimated using diffusion-weighted images, i.e. large portions of tissue defined as permanently damaged are still viable. Reduced diffusion can lead to exaggerating of the size of the infarct core; while the perfusion weighted images were seen to lead to exaggerating the size of the penumbra by incorporating benign oligemia [5, 6]. The deviations can be large when comparing the size of ischemic core determined via reduced apparent diffusion coefficient (ADC) to the accepted reference standard of triphenyltetrazolium chloride (TTC) staining [7]. Furthermore, ADC values can reverse in diffusion lesions from low during acute ischemia to high during the subacute stroke phase [8, 9]. Since the region of altered diffusion can include viable tissue during the acute phase, the validity of the perfusion–diffusion mismatch approach has been questioned. It is believed that previous clinical trials failed to show benefits of MRI-based thrombolysis when the diffusion–perfusion mismatch was applied as a selection criteria [10] or are ongoing (WAKE-UP Trial or ECASSIV Trial). To overcome these limitations we have to develop novel imaging strategies [11] in order to better define the viable tissue. One new approach to improve assessment of permanently-damaged and still-viable ischemic tissue fractions is to add ^23^Na-MRI to the existing MRI protocol during the acute and non-acute stroke phase.

As opposed to the rapid change of diffusion coefficient after stroke onset, the ^23^Na signal in patients can has been reported constant for up to 32 h in penumbra and up to 7 h in core tissue in areas defined as hypoperfused stroke tissue [12]. More interestingly, tissue sodium concentration (TSC) changes were quantified in the penumbra and core in a pre-clinical model of stroke. More recently, sodium signal has been measured below contralateral values during the acute stroke phase in penumbra [13]. Thus, the assumption that TSC increases immediately after arterial occlusion in still-viable stroke tissue [14] must be revised considering recent reports of ^23^Na signal as measured in in vivo stroke models and stroke patients. In an experimental in vivo model of myocardial infarction it has been shown that intracellular sodium is a sensitive marker for cellular bioenergetics failure. Reperfusion of the myocardium caused a decrease in total sodium MR image intensity, which indicates that TSC can potentially serve as a marker for myocardial viability [15].

We hypothesized that combined ^1^H- and ^23^Na-MRI of acute and non-acute stroke can improve delineation between reversible and irreversible ischemic injury. This study will use the reperfusion model of the stroke in order to identify the penumbra. For the first time, sodium and ADC variations before and after reperfusion will be investigated in order to predict the penumbra area in the early phase in an in vivo model of transient stroke.

## Methods

The section is in parts identical to previous publication by Wetterling et al. [20] where the methodology of acquiring sodium MRI data as well as perfusion and diffusion MRI data was described with a main focus on the rf coil technology and a single case example. This study describes the clinical multi-sample study and results in more detail with a focus on the preclinical and clinical interpretation.

### Ethics

All experiments were carried out in strict accordance with the guidelines for the European Community Council Directive (2010/63/EU) for Protection of Vertebrate Animals Used for Experimental and other Scientific Purposes and were approved by the Animal Ethical Committee for Laboratory Animal Experiments at the Regional Council in Karlsruhe, Germany (Licence No. 35-9185.81/G-176/10). The study complies with the ARRIVE guidelines (Animal Research: Reporting In Vivo Experiments).

### Middle cerebral artery occlusion

Transient middle cerebral artery occlusion (tMCAO) was induced in male Wistar rats by using the intraluminal filament technique previously described [16, 17]. Briefly, anesthesia was induced using 4.5% isoflurane in N_2_O:O_2_ (70:30) and then maintained by inhalation of 1.5–2% isoflurane in N_2_O:O_2_ (70:30) during the surgical procedure. A rectal temperature probe connected to a homoeothermic blanket was inserted for maintenance of a body temperature of 37 °C during the operational procedure. A polyethylene catheter was placed in the tail artery for measurement of blood pressure, pH, pCO_2_, pO_2_ and blood glucose prior to the occlusion. An incision was made in the midline of the neck, and the right common, internal and external carotid arteries were exposed. The right common and external carotid arteries were ligated. The common and external carotid arteries were permanently ligated by sutures. A silicon rubber-coated monofilament (Doccol Corporation, MA, USA) was inserted into the internal carotid artery via an incision in the common carotid artery, and further advanced until the rounded tip reached the entrance of the right middle cerebral artery. To confirm a proper occlusion of the right middle cerebral artery, a laser-Doppler probe (Moor Instruments, United Kingdom) was fixed on the skull (1 mm posterior to the bregma and 6 mm from the midline on the right side) measuring regional cortical blood flow. The resulting occlusion was confirmed by laser-Doppler flowmetry that showed an abrupt reduction of cerebral blood flow (CBF) of at least 70 ± 10%. After fixing the filament all wounds were closed and the rat was placed into the magnet bore. The occlusion time for the MCAO was 90 min, after which the filament was carefully withdrawn and the rat was placed into the scanner again. The reperfusion was monitored by the laser Doppler by obtaining an increase of the CBF of about 70 ± 10%. At the end of the surgery, the rats received a subcutaneous injection of 10 ml of isotonic saline for hydration.

### Pre-clinical multinuclear ^23^Na/^1^H MR imaging protocols at 9.4 T

The anesthesia was maintained by inhalation of 1.5–2% isoflurane in N_2_O:O_2_ (70:30) during the imaging procedure. The breathing rate and body temperature were monitored during the imaging procedure. A 9.4 T preclinical MRI system (Biospec 94/20, Bruker, Germany) equipped with 740 mT/m gradients was used with a specifically developed double-tuned ^1^H transmit-only receive-only and ^23^Na transceiver radio-frequency (rf) resonator system [18]. The imaging was performed accordingly to previous described protocols [18]. The global *B*_0_-shim within the field-of-view of the ^1^H receive-only surface resonator, center frequency and rf-reference pulse voltage was determined automatically at the ^1^H frequency prior to acquiring three ^1^H localizers in coronal, axial, and sagittal views for ^1^H slice positioning. The first MRI scanning was ^1^H angiography, followed by ^1^H diffusion-weighted and ^1^H spin-echo imaging with various TEs. In addition an arterial spin labeling experiment was performed to obtain perfusion weighted ^1^H MR Images. Prior to ^23^Na imaging *B*_*0*_ was shimmed manually within the imaging field of view before the center frequency was adjusted and ^23^Na MR imaging data was acquired.

The ^1^H localizer were acquired with the following 2D-gradient echo sequence parameters: 1 slice, slice thickness (ST) = 2 mm, field-of-view (FOV) = (80 × 80) mm^2^, nominal in-plane resolution = (0.625 × 0.625) mm^2^, TR/TE = 100/6 ms, flip angle (FA) = 30°, bandwidth (BW) = 50 kHz/FOV, and acquisition time (TA) = 13 s.

### Apparent diffusion coefficient

^1^H diffusion-weighted imaging was acquired by previously described protocol [18]. The 2D echo planar imaging (EPI) with three diffusion directions, two different *b*-values (530 and 1079 s/mm^2^), and five unweighted *b* = 0 s/mm^2^ acquisitions, 10 slices, ST = 1.8 mm, inter slice distance = 2 mm, FOV = (25 × 30) mm^2^, nominal in-plane resolution = (0.31 × 0.31) mm^2^, TR/TE = 4000 ms/ 21.3 ms, BW = 357 kHz/FOV, and TA = 88 s.

### Sodium

For ^23^Na MRI with high spatial and temporal resolution 3D FLASH was used. The sequence parameters used are previously described [18]; FOV = (64 × 64 × 128) mm^3^, nominal voxel resolution after two-fold 3D zero-filling = (0.5 × 0.5 × 2) mm^3^, TR/TE = 21/2.9 ms, 10% partial echo acquisition, BW = 4 kHz/FoV, rf pulse (gauss) length = 0.1 ms. The Ernst angle was adjusted to be within the striatum region of the brain, TA = 4 min 57 s TA. Previously, a 3D CSI technique was used at the 9.4 T to observe the T_2_^*^ decay in mouse brain tissue which can be regarded similar to rat brain tissue in terms of MRI relaxation parameters. The decay was well described by a single-exponential transversal relaxation time of 5–9 ms. Therefore, a TE of 2.9 ms was deemed to be short enough to measure the qualitative change of the sodium MRI signal after ischemic stroke in this study.

### T_2_-weighted

^1^H T_2_ maps were reconstructed from multi slice multi echo (MSME) sequence data acquired as follows: 3 axial slices, ST = 2 mm, inter-slice distance = 4 mm, FOV = (64 × 64) mm^2^, nominal in-plane resolution = (0.5 × 0.5) mm^2^, TR = 2888 ms, TE = 11–176 ms in 16 increments, BW = 60 kHz/FOV, TA = 4 min 37 s.

### Angiography

To record angiographic images of the cerebral artery system 3D ^1^H Time-of-Flight (ToF) sequence was used with following parameters: FOV = (40 × 40 × 40) mm^3^, nominal voxel size = (0.16 × 0.16 × 0.31) mm^3^, TR/TE = 15/2.5 ms, BW = 100 kHz/FoV, FA = 20°, and TA = 6 min 8 s.

### Cerebral blood flow

^1^H perfusion-weighted imaging was obtained through FAIR imaging scheme with spin-echo RARE sequence. The acquisition parameters were as described in previous study [18]. In brief, ST = 2 mm, inversion slab thickness = 5 mm, nominal in plane resolution = (0.9 × 0.9) mm^2^, FOV = (11.52 × 11.52) cm^2^, TR/TE = 18,000/56 ms, recovery time = 10 s, RARE factor = 72, BW = 75 kHz/FOV, inversion time (IR) = 2000 ms and TA = 1 min 39 s.

### Histological examination

All animals were euthanized 24 h after reperfusion and fixated in 4% formalin overnight. Postmortem, 10 µm coronal cryosections were cut at 400 µm intervals and stained with hematoxylin–eosin–saffron (H&E) staining accordingly to previous protocols [19].

### Data analysis

Pre-clinical rat brain images were analysed using a self-written script in Matlab^®^ (The Mathworks, Natick, MA). The fourth stereotaxic coronal level covering the center slice of the rostrocaudal extent of MCA territory (8.2 mm from the interaural line) was used to carry out noninvasive ADC, and sodium measurements on a single slice. ROIs were selected manually in ipsilateral and contralateral tissue in ADC maps, and ^23^Na images at each time point.

**Fig. 1:**
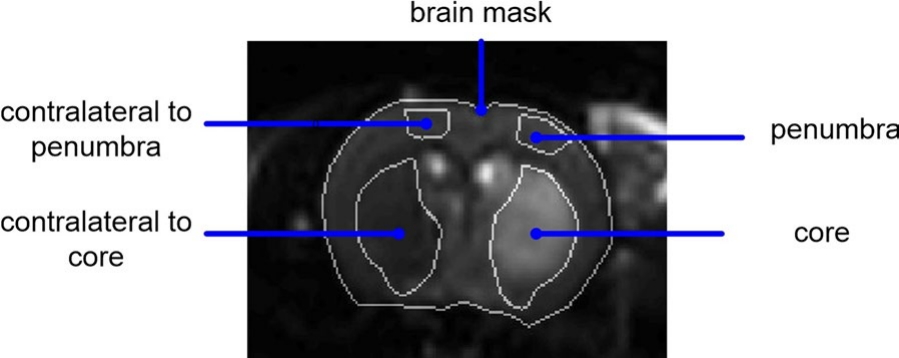
Example of how region-of-interest (ROI) for penumbra, core tissue and contralateral side was selected. The ROIs on the contralateral side was used to normalize the measured signal values. By normalizing all signals to contralateral ROIs reduces the influence of the coil profile on the measurement results

Core tissue was defined as an area with <57% perfusion [4, 20] during the acute phase before reperfusion and with elevated T_2_ signal during the sub-acute phase compared to the same area in the contralateral hemisphere. This value was defined as the one where the increase of the T_2_ signal is significantly higher than the variation observed in the healthy tissue (contralateral). An example of a T_2_ image chosen for core definition is graphed in Fig. 3 together with the corresponding histology slice. The core region was manually drawn on the outline of the high intensity T_2_ image area. Penumbra was considered to be the mismatch area between the hypoperfused area and the core. ROIs were positioned bilaterally on the MRI maps and images encompassing the core and the penumbra. The location of the ROIs varied in between animals dependent on the lesion extent. To ensure that tissue of similar tissue fate (i.e. core or penumbra) core was selected in areas that covered the somatosensory cortex in some animals while in other animals core was confined to the caudate putamen. All the ROIs were selected manually and drawn carefully with reference to anatomic images and a stereotaxic rat brain atlas. Figure 1 illustrates an example how the region of interest was selected.

**Fig. 2:**
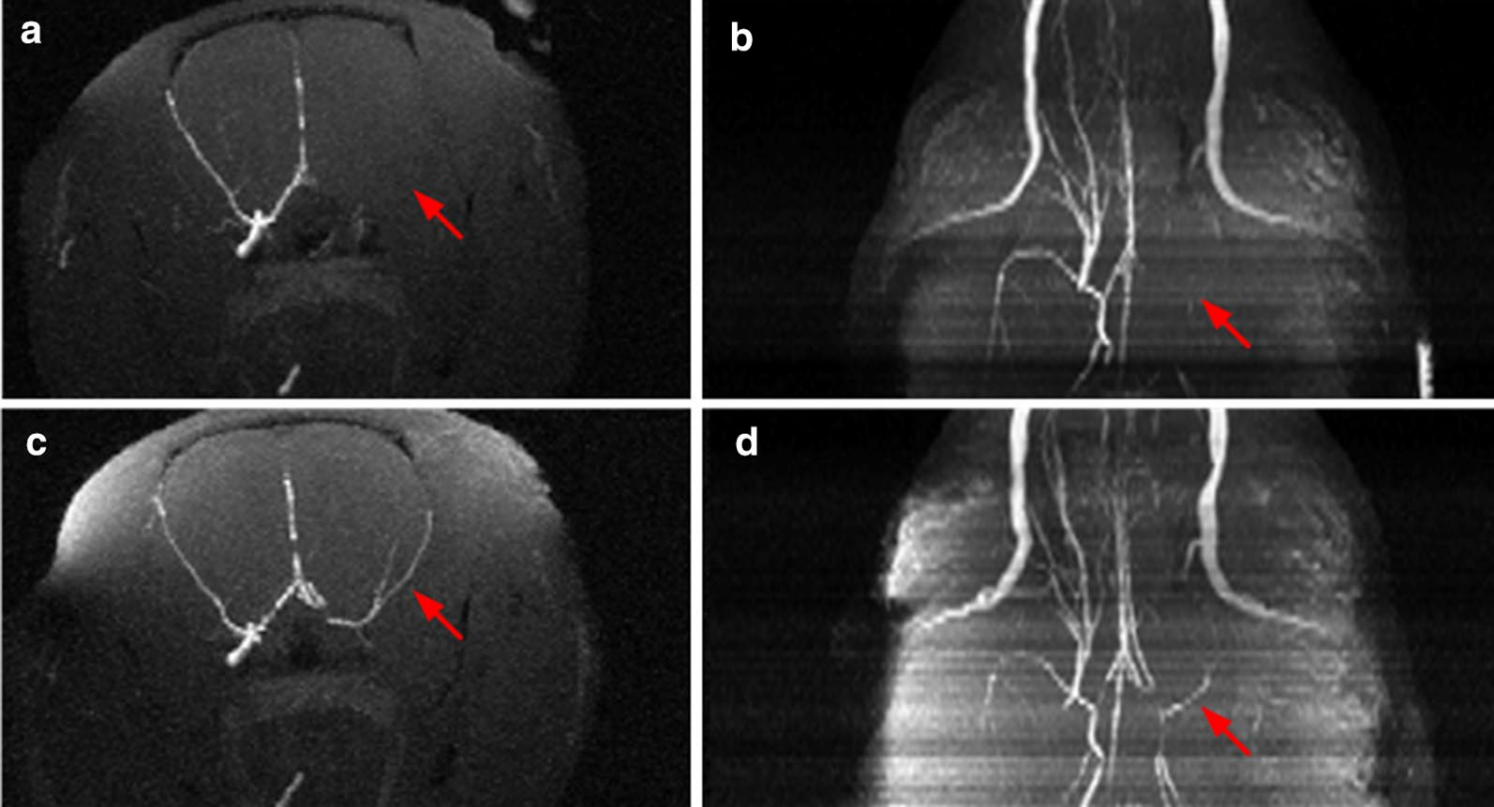
Representative example of the maximum intensity maps reflecting angiographic information before (**a** coronal section, **b** transversal section), and after reperfusion (**c** coronal section, **d** transversal section)

The ratio of contralateral to ipsilateral signal intensities were computed from measurements taken in the respective core and penumbra areas in the sodium MR image and ADC map. Data are expressed as mean ± standard error of the mean (SEM), and n refers to the number of rats. Statistical analyses were performed using a paired student t-test, where p < 0.05 was considered significant. Animal number 1, 2, 3, 7, 9, and 10 were scanned at all three data points while animal number 4, 5, 6, and 8 were only scanned before and after reperfusion, due to mortality. Angiographic images were used to confirm proper blockage and reperfusion of MCA (Fig. 2).

## Results

There was no difference between the animals for physiological parameters which were all maintained within physiological ranges. In total 10 animals were included in the study, 4 of them died before the third measurement time point at 24 h after stroke onset time. The high mortality rate is due to extended anaesthesia to be able to perform the MRI direct after occlusion and subsequently after reperfusion. The rat was under anesthesia in total for approximately 3–4 h to be able to perform all measurements.

The MCA was successfully occluded and reperfused in all animals (n = 10) as observed from the non-existing right MCA signal in the maximum intensity map before reperfusion (Fig. 2a, b) and the recovery of the MCA signal after filament removal (Fig. 2c, d). These results were also confirmed by the cerebral blood flow values observed by laser-Doppler flowmetry during occlusion there was a reduction of the cerebral blood flow of 70.6 ± 4.4% compared to baseline 100%. During the withdrawal of the filament the cerebral blood flow increased 71.3 ± 3.8% compared to baseline which was set to 0%

### Cerebral blood flow

**Fig. 3:**
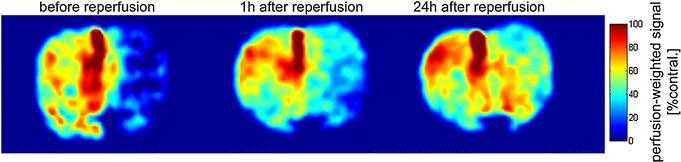
Representative example of the perfusion weighted images acquired before, after, and 24 h after reperfusion: coronal section at the level of the Caudate–Putamen. Perfusion improved after reperfusion, however, a clear deficit in the contralateral side remained, which improved 24 h after reperfusion

**Fig. 4:**
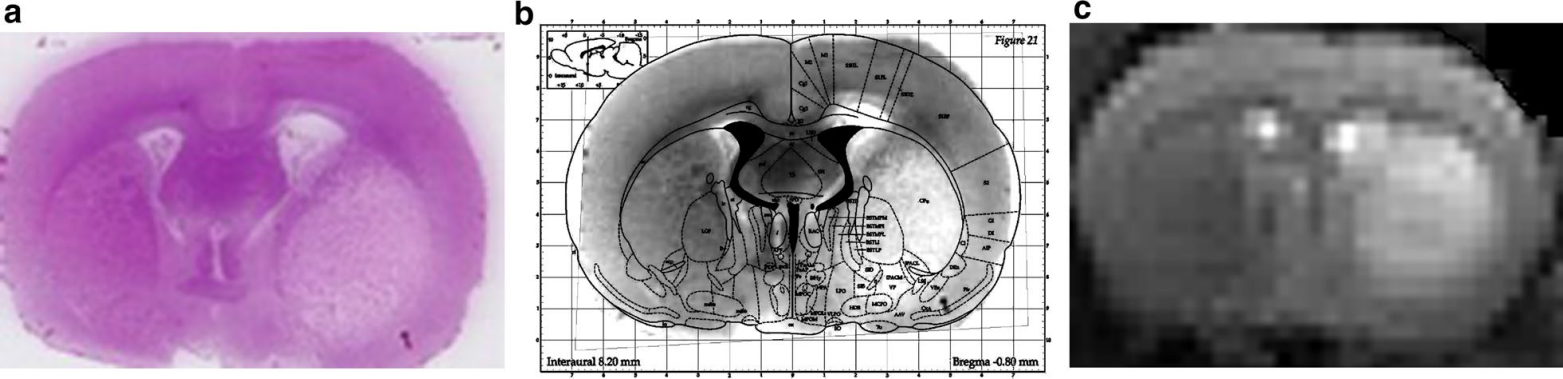
Representative image of histology (**a**) superimposed with corresponding slice from anatomical atlas (**b**) showing a clear damage in caudate putamen, but no damage in upper and lower cortex. T_2_-weighted image at 24 h (**c**) after stroke confirming the identical lesion via hyperintense signal intensity

The lack of arterial blood flow resulted in hypoperfusion of the right hemisphere as shown in the perfusion-weighted images acquired before reperfusion (Fig. 3a). Perfusion was improved after arterial reperfusion, but a clear deficit in the right hemisphere remained (Fig. 3b) which improved at 24 h after reperfusion (Fig. 3c). Penumbra measured with perfusion weighted image extended across motor cortex (MC), primary somatosensory cortex (PSC), and secondary somatosensory cortex (SSC). Core tissue characterized by an increase of the T_2_ signal intensity (see “Data analysis” section) grows across time and concerned caudatoputamen (CPu) in most cases and rarely extended to SSC and PSC (Fig. 4b).

### Histology and correlation with MRI

The lesion in the T_2_-weighted image at 24 h after reperfusion correlates with the lesion from H&E staining at 24 h after reperfusion. A clear area of damage is observed in the caudate putamen, but no damage is observed in upper and lower cortex. Figure 4a illustrate the histology with the corresponding slice from the T_2_-weighted image (Fig. 4c) for one representative rat.

### Apparent diffusion coefficient

**Fig. 5:**
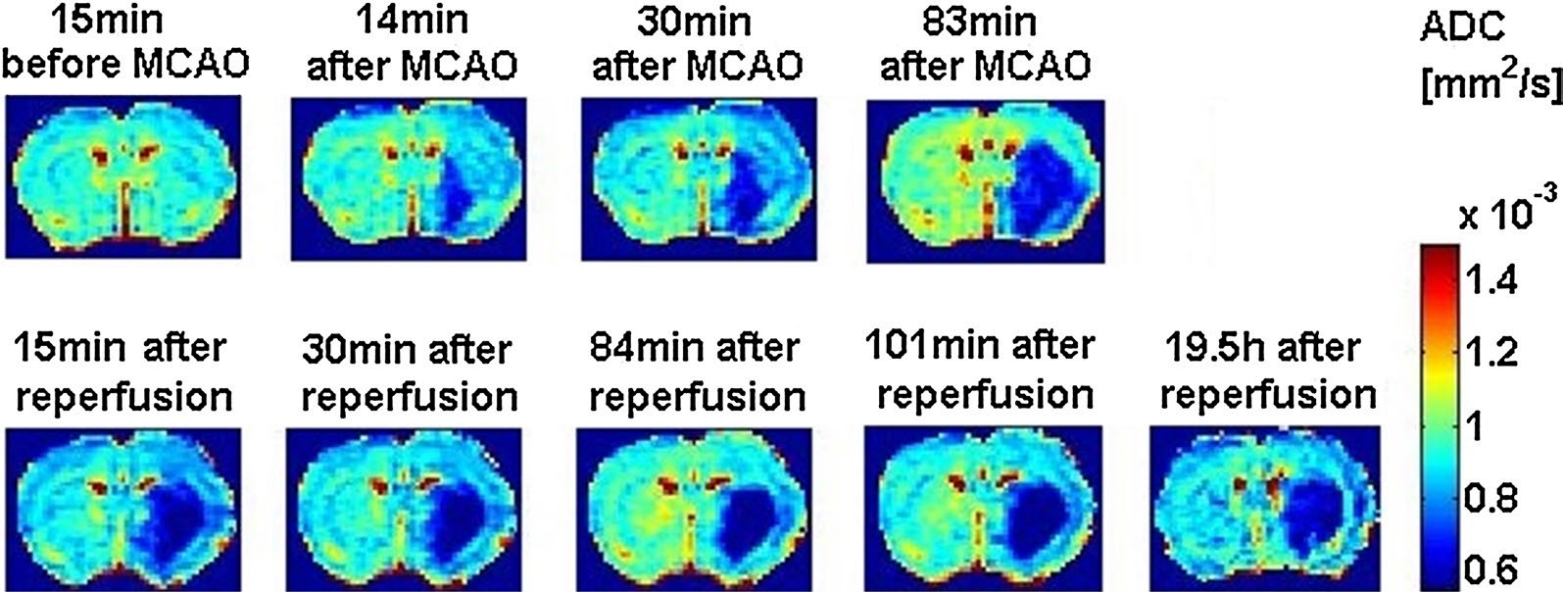
Apparent diffusion coefficient (ADC) maps for one representative rat before (*first row*), and (*second row*) after reperfusion. Note the recovering ADC in the lower cortex after reperfusion

The ADC signal reduced nearly in the entire hypoperfused area including the cortex, although the final lesion size was restricted to the caudate putamen (Fig. 5). The ADC lesion after reperfusion matched the final lesion size well within the caudate putamen. At 24 h after reperfusion the ADC lesion visually vanished, although a decrease in ADC compared to contralateral was still measured, yet this decrease was above the commonly applied viability threshold. The quantitative results for the tMCAO experiment are summarized in Table 1. The qualitative observations made for each individual stroke matched the group results. ADC was significantly reduced for all time points in core tissue (Table 1) while ADC was significantly higher at 24 h after reperfusion compared to before reperfusion in core (Table 1). More interestingly, ADC was decreased by 5–10% in penumbra.

**Table 1:** ADC and sodium measurements from core and penumbra before, after and at 24 h after reperfusion

	Before reperfusion (N = 10)	After reperfusion (N = 10)	24 h after reperfusion (N = 6)
Sodium penumbra (%contr.)	88 ± 14*	98 ± 11 (p = 0.528)	105 ± 16
Sodium core (%contr.)	101 ± 8	123 ± 14*	168 ± 27*
ADC penumbra (%contr.)	91 ± 7*	95 ± 5*	96 ± 3*
ADC core (%contr.)	68 ± 7*	67 ± 15*	80 ± 10*

Data are expressed as mean ± SEM

* Significant difference between contralateral and ipsilateral side (p ≤ 0.05) indicate whether sodium or ADC differed from contralateral measurements

### Sodium

**Fig. 6:**
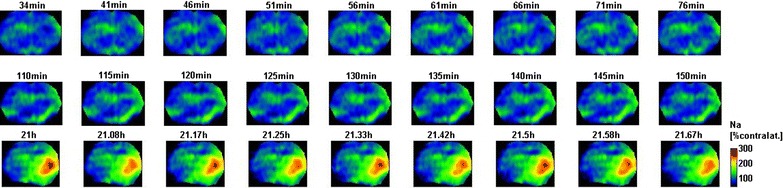
Representative example of sodium MR images acquired before (*first row*), after (*second row*), and 1 day after reperfusion (*third row*)

Sodium was low in penumbra before reperfusion, but seemed to recover after reperfusion with a significantly higher sodium signal measured at 24 h after reperfusion compared to before reperfusion, while the actual sodium signal was not significantly different from contralateral sodium signal after reperfusion. In core, sodium was significantly increased after reperfusion, but similar to contralateral sodium before reperfusion (Fig. 6).

**Fig. 7:**
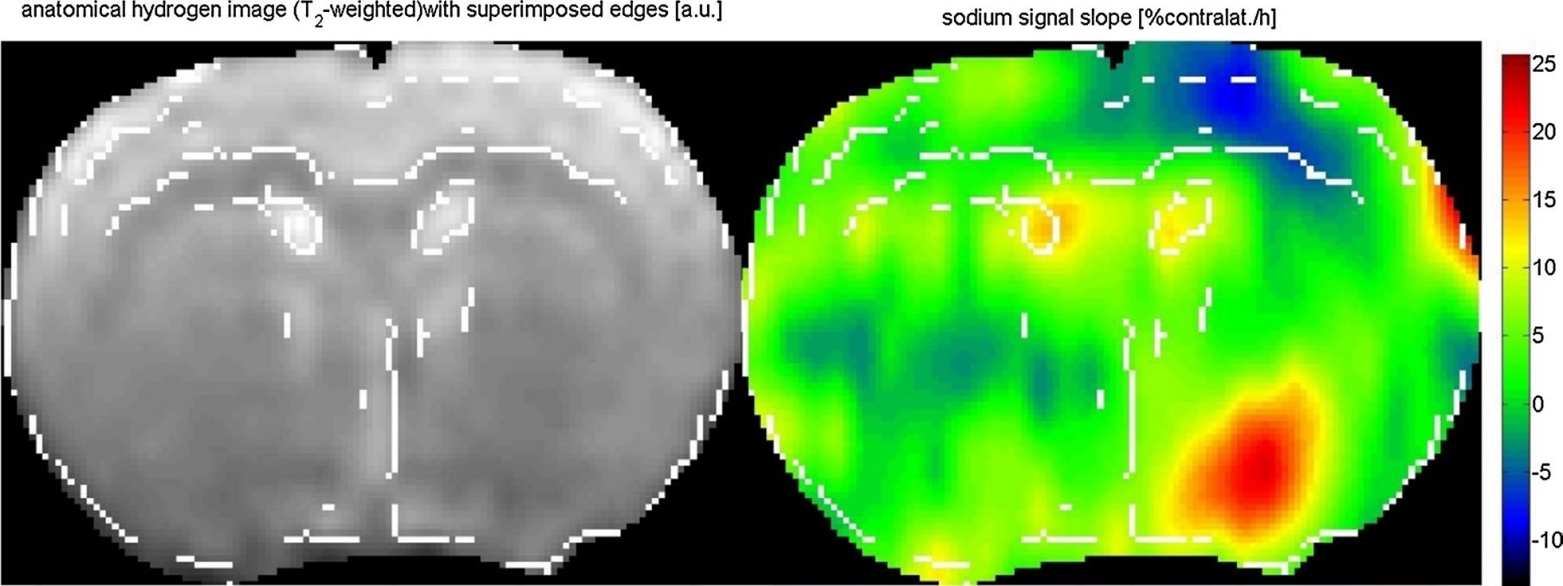
Sodium slope map before reperfusion (15–80 min after MCAO, 9 time samples normalized to contralateral caudate putamen tissue) indicating a strong increase in sodium signal in subcortical tissue and a strong decrease of sodium signal in upper cortex. *Red* indicates that the sodium signal increased at a rate of 20%/h, while *blue* indicates that sodium signal decreased at a rate of 10%/h over the observed time range of 15–80 min after MCAO

The sodium slope map before reperfusion indicate a strong increase in sodium signal in subcortical tissue and a strong decrease of sodium signal in upper cortex. The sodium signal increased at a rate of 20%/h while the sodium signal decreased at a rate of 10%/h (Fig. 7).

## Discussion

For the first time ^23^Na-MRI was assessed in penumbra and core tissue during and after transient cerebral ischemia in a pre-clinical model of stroke. Lin et al. recorded sodium measurements during tMCAO in rats at later than 12 h after stroke onset time. Due to the lack of perfusion MRI in their study, the variations of sodium, and ADC have never been studied during the acute stroke phase nor were they able to differentiate the signals for core and penumbra tissue [7]. The data recorded in this study provided very high spatial resolution possible due to specialized coil technology [20]. Hence, in this study substructures within the stroke lesion such as presumed core and penumbra could be analysed. This is in contrast to previous studies, which only allowed investigation of core tissue in monkeys during the acute phase [21]. Contrary to the present hypothesis by several research groups that ^23^Na-MRI can increase above normal values after ischemia in still viable tissue [22] we demonstrated that ^23^Na was below contralateral values during 90 min tMCAO in penumbra. Increasing ^23^Na MRI signal was detected in penumbra (up to normal contralateral values) and in core (by 60%) after arterial reperfusion. Sodium signal may indeed reduce or remain at normal levels in still viable tissue.

The sodium changes observed using sodium MRI have been related to cellular volume and compartmental concentration variations elsewhere [13, 18]. Within minutes after occlusion an ischemic cascade starts, which include sodium–potassium pump failure. Depletion of oxygen and glucose, leads to decrease in adenosine triphosphate (ATP) which leads to impairment of the sodium–potassium pump. Dysregulation of sodium–potassium pump will provoke a loss of sodium homeostasis and therefore increase of intracellular sodium concentration, which will lead to cellular edema (i.e. swelling) [13, 18] and if energy deprivation persists this will ultimately lead to cell death [23, 24]. Most living cells have a high concentration of intracellular potassium and a low concentration of intracellular sodium [25]. During the early hours of ischemic stroke cytotoxic cerebral edema occurs in the presence of an intact blood brain barrier (BBB). The ATP depletion and disturbance of intra-extracellular Na^+^ transportation are responsible for the cytotoxic edema. As the ischemia progresses, the BBB begins to breakdown and ions and water move paracellularly from blood into brain causing vasogenic edema. During this process the brain Na increases which our results also demonstrates. During the cytotoxic edema process there is an accumulation of water in the cells and the T_2_ increases but the ADC value decreases [26]. In our study we observe the similar as previous studies, that the ADC values decreases during the cytotoxic edema (before reperfusion) and during the vasogenic edema which results from endothelial disruption both the T_2_ and ADC values increases as observed after reperfusion during the subacute phase.

We would like to point out that MRI cannot provide sufficient detail to differentiate microscopically between cell types (e.g. astrocytes and other brain cells) nor was the functionality of the blood brain barrier observable with the chosen approach. However, MRI can reveal macroscopic measurements in tissue non-invasively and with sufficient temporal resolution in one and the same animal. Moreover the herein presented measurements provide volumetric information with no differentiation between intra- and extracellular spaces. The non-invasiveness and the ability to resolve tissue degrading processes in vivo come at the expense of lack of cellular granularity. However, we would like to highlight that the temporal evolution in different parts of the stroke tissue can be vastly different with some tissue being entirely damaged after 90 min as indicated by elevated total sodium concentration (indicating cell membrane rupture). Other tissue would only reach that state after 6 h and reperfusion then adds many more variations to potential tissue fate.

In our study, we showed a reduced sodium signal which is suggested to be the penumbra. A decrease of sodium measurement before reperfusion characterizes the penumbra, whereas an increase of sodium measurement after reperfusion characterizes the core of the ischemic area. After acute ischemia and reperfusion an increase in Na^+^ MRI intensity was demonstrated to be associated with nonviable tissue [27]. The exact mechanism behind the low sodium in the penumbra before reperfusion remains unclear. One possible explanation would be that it is related to a transient reversed mode of Na^+^/Ca^2+^ exchanger (NCX) activity during the ischemia resulting in sodium efflux and calcium influx [28]. After reperfusion the NCX work in a forward mode, which can explain the increased sodium. Experimental studies have demonstrated that activation of NCX to work in reversal prevents neuronal damage and death [29].

After an ischemia Na^+^ will increase because of inhibition of the sarcolemmal Na^+^, K^+^-ATPase and increased influx of Na^+^ channel via the Na^+^–H exchanger [30, 31]. ADC measurement revealed a decrease in all the ischemic area with a stronger decrease in the core.

Although parts of the finally damaged tissue showed early low ADC (with a decrease of above 30%, see Table 1), it remains unclear whether low ADC occurs long before tissue viability loss or indeed at the incidence of permanent tissue damage. A premature decrease in ADC may also explain the discrepancy in ^23^Na and ADC lesion during the acute phase. An early ADC decrease may prevent some patients from receiving treatment if presenting with no diffusion/perfusion mismatch and hence no presumed penumbra despite the fact that the entire tissue or at least larger fractions of the tissue could have still been salvaged via thrombolytic therapy.

These results highlight the power of combined ADC and sodium measurements to assess vulnerable ischemic tissue. In areas of low sodium, recovery may be monitored while ADC is more or less unchanged. This may be a useful marker to monitor tissue functionality during the rehabilitation stages when clinical deficits may still be apparent, e.g. restricted movement capability despite no apparent cortical tissue damage. Hence, combined sodium and ADC measurements may not only prove useful as a time independent marker for tissue damage, but also as a marker of tissue recovery during therapy.

ADC has been suggested to be a good marker for tissue viability loss. The dynamics of the ADC however lead to a spontaneous recovery during the sub-acute and chronic phase making it difficult to assess tissue viability without precise knowledge of stroke onset time. Sodium MRI provides a viability marker that does not reverse. Hence, ADC measures combined with sodium MRI may be a good approach to assess tissue viability in stroke.

## Conclusions

Reduced sodium-MRI signal in conjunction with reduced ADC may serve as a viability marker for penumbra detection and can complement hydrogen diffusion- and perfusion-MRI in order to facilitate time-independent assessment of tissue fate in stroke patients. The results of this study may contribute to the development on new clinical diagnostic tool for early detection of ischemia and of the penumbra.

## Abbreviations

ADC: apparent diffusion coefficient
ATP: adenosine triphosphate
BW: bandwidth
CPu: caudatoputamen
CBF: cerebral blood flow
H&E: hematoxylin–eosin–saffron
FA: Flip angle
FOV: field-of-view
IR: inversion time
MC: motor cortex
MSME: multi slice multi echo
MRI: magnetic resonance imaging
NCX: Na^+^/Ca^2+^ exchanger
rf: radio-frequency
SSC: secondary somatosensory cortex
ST: slice thickness
TA: acquisition time
ToF: Time-of-Flight
tPA: tissue plasminogen activator
TSC: tissue sodium concentration
tMCAO: transient middle cerebral artery occlusion
